# Pure PBL, Hybrid PBL and Lecturing: which one is more effective in developing cognitive skills of undergraduate students in pediatric nursing course?

**DOI:** 10.1186/s12909-018-1305-0

**Published:** 2018-08-10

**Authors:** Mohsen Salari, Amrollah Roozbehi, Abdolvahed Zarifi, Rohani Ahmad Tarmizi

**Affiliations:** 1 Nursing Department, School of Nursing, Yasuj University of Medical Sciences, Yasuj, Iran; 2 Education Development Center, School of Medicine, Yasuj University of Medical Sciences, Yasuj, Iran; 3 English Language Department, Faculty of Humanities, Yasouj University, Yasouj, Iran; 4 Faculty of Educational Studies, University Putra Malaysia, Serdang, Selangor Malaysia

**Keywords:** Problem-based learning (PBL), Cognitive skills, Pediatric nursing course

## Abstract

**Background:**

Nursing education in Iran has conventionally focused on lecture-based strategies. Improvements in teaching and learning over the years have led to an expansion of the pedagogies available to educators. Likewise, there has been a suggestion for a move toward more learner-centered teaching strategies and pedagogies that can result in improvement in learning. This study was undertaken to investigate the effects of Problem-Based Learning in developing cognitive skills in learning Pediatric Nursing among university students.

**Methods:**

In this quasi-experimental, posttest-only nonequivalent control group design, the subjects were undergraduate students who had enrolled in Pediatric Nursing II at Islamic Azad University in Iran. The experiment was conducted over a period of eight weeks, one two-hour session and two two-hour sessions.

Two experimental groups, Pure Problem-Based Learning (PPBL) and the Hybrid Problem- Based Learning (HPBL), and one Lecturing or Conventional Teaching and Learning (COTL) group were involved. In the PPBL group, PBL method with guided questions and a tutor, and in the HPBL group, problem-based learning method, some guided questions, minimal lecturing and a tutor were used. The COTL group, however, underwent learning using conventional instruction utilizing full lecture. The three groups were compared on cognitive performances, namely, test performance, mental effort, and instructional efficiency. Two instruments, i.e., Pediatric Nursing Performance Test (PNPT) and Paas Mental Effort Rating Scale (PMER) were used. In addition, the two-Dimensional Instructional Efficiency Index (IEI) formula was utilized. The statistical analyses used were ANOVA, ANCOVA, and mixed between-within subjects ANOVA.

**Results:**

Results showed that the PPBL and HPBL instructional methods, in comparison with COTL, enhanced the students’ overall and higher-order performances in Pediatric Nursing, and induced higher level of instructional efficiency with less mental effort (*p* < 0.005). Although there was no significant difference in lower-order performance among the groups during the posttest (*p* = 0.92), the HPBL group outperformed the COTL group on the delayed posttest (*p* = 0.028).

**Conclusions:**

It may be concluded that both forms of PBL were effective for learning Pediatric Nursing. Moreover, PBL appears to be useful where there are shortages of instructors for handling teaching purposes.

**Electronic supplementary material:**

The online version of this article (10.1186/s12909-018-1305-0) contains supplementary material, which is available to authorized users.

## Background

Nursing students encounter many clinical challenges in current healthcare atmosphere [1, 2]. It is unfortunate that they often have no prior experience to deal with these varying complex situations. More importantly, traditional teaching methods often fail to enable students to cope with these clinical conditions [3]. Subsequently, these concerns call for an alternative approach with higher efficacy in teaching nursing.

Lecturing is the most common teaching strategy in the nursing education, and problem based learning has not yet been of widespread use in Asia [4, 5]. In the current nursing educational context of Iran, the main focus of almost all the universities for nursing education is on the traditional teacher-based approach [6]. On the other hand, it is often observed that the most important barriers to the implementation of student-centered strategies in Iran are students’ lack of familiarity with the new strategies, inadequate skills in group work and active interaction, and the large number of students in a class [7]. This is the case despite the fact that the world today needs graduates who can take advantage of their own diverse skills and in-depth or upper level academic knowledge in order to benefit from professional problem solving and life-long learning. So, a comprehensive curriculum should be accommodated to the future profession and offers students learning opportunities to acquire problem solving skills so that they can improve the healthcare systems they work with through appropriate decision making [5, 8–11]. It is essential for nursing educators to apply appropriate teaching methods to improve the students’ performance in clinical nursing [12, 13].

It is often argued that development, learning, and higher mental functions take place through social interactions in a constructive environment by engaging in an active process of discovering knowledge [14–17]. Also, student-centered strategies can enable the educators to help learners to be actively involved in promoting lifelong learning, problem solving, critical thinking, group process skills, creativity, information literacy, student success, and empowerment [18, 19]. Likewise, students tend to prefer new approaches if they are more enjoyable, have potentiality to grow enthusiasm and interest in students towards the course and its content [20, 21], enhance interactions between students and their instructors, and increase students’ understanding of the course content [22, 23].

Likewise, educators need to serve as a facilitator or apply a floating tutoring role in the context of small groups. Such a role is uncommon in traditional educational approaches. One strategy in which the attention shifts from teacher to student is Problem-Based Learning (PBL). This strategy is able to get the graduates ready for the uncertainties of future managerial practice and facilitates the students’ construction and reconstruction of their own knowledge base [12]. In PBL, educators are full partners in the learning process and not the major holder of knowledge [24]. Some of the related studies show that PBL can encourage students to learn better and foster the retention and lifelong learning skills that can be transferred into clinical practice and finally increases the quality of patient care [18, 25–27]. PBL could help to bridge the gaps between education, practice, and knowledge development in professional schools including nursing, which is, in turn, able to prepare the learners for their future role as Registered Nurses [28].

PBL is highly flexible, and it can be used in different ways and in different contexts [29, 30]. Some settings use the Traditional or Pure PBL strategy; some others tend to modify the strategy by incorporating some traditional techniques. The results of these modifications can improve the educational process for learners and instructors, or result in unsuccessful attempts and a passing trend [31]. There are cases of institutions that have successfully implemented PBL as a hybrid curriculum combined with other learning strategies like lectures, practical classes, etc. [32, 33]. In these contexts, educator, as a facilitator, strives to guide the students. It is often argued that, the idea of scaffolding in the zone of proximal development and the technique of facilitating PBL groups are complementary processes [34]. This is because scaffolding takes shape through guidance, and develops faster to link students’ existing abilities with their intended goals [35]. This can result in a reduction of the learners’ cognitive load to easily solve the problems which need high mental effort [36].

It has been more than 40 years that PBL, as a student-centered strategy, has been substituted for traditional ones, and there exists some empirical research evidence in support of problem-based learning [22, 33, 37–39]. Nevertheless, some research findings and meta-analyses have revealed a number of gaps in the PBL literature. For instance, the results were mixed and the performance on the exams of the fundamental courses of the students experiencing PBL strategies appeared to be lower than that of the students exposed to conventional approaches [5, 40–43]. Also, little has been done to reveal what exactly takes place in a PBL class to nurture the necessary skills. Moreover, to the best of our knowledge, no research has been carried out to compare the outcome of the application of Pure Problem-Based Learning (PPBL) and Hybrid Problem-Based Learning (HPBL) strategies in teaching Pediatric Nursing (PN) course. In addition, no research has yet been done on the relevance of cognitive load theory and instructional efficiency in nursing education in Iran [7].

The purpose of this study is, therefore, to investigate the cognitive effects of PPBL, HPBL, and Conventional Teaching and Learning (COTL) methods on learning Pediatric Nursing.

## Methods

### Study design

This study has adopted a quasi-experimental posttest-only nonequivalent control group design to examine the effects of PBL instructional strategies on measures of performance, mental effort and instructional efficiency.

### Participants, setting and measures

The accessible population in this study consisted of all the junior nursing students who attended the course Pediatric Nursing (PN) II in two branches of Islamic Azad University (IAU) in Yasuj and Gachsaran, Kohgiluyeh-BoyerAhmad (KBA) province, Iran.

The instructional strategies such as PPBL, HPBL, and COTL as the independent variables and performance, mental effort and instructional efficiency as the dependent variables were considered. In addition, threats to internal and external validity were taken into account and controlled. It should be pointed out that the experiment was conducted over a period of 8 weeks.

### Inclusion criteria

The participants were junior nursing students who had passed Pediatric Nursing I. They, however, had not received any training on problem-based learning before the main experiments.

### Exclusion criteria

The exclusion criteria in the study were missing two or more class sessions, refusing to take one of the tests administered, and failing to complete the questionnaires. It should be pointed that no participant appeared to have these criteria.

### Baseline test

Since all the participants had already passed the PN (I) as a prerequisite course, their PN (I) scores were compared to make sure that they had similar prior PN knowledge. Likewise, the students’ scores of Prior Performance Test on the three topics of PN course which had been taught before the experiment were used as covariate to ascertain that the students in all three groups were similar in their PN course knowledge.

### Sampling

The sampling method was multistage cluster. At first, one of the regions of all the 13 regions of IAU was selected purposively. Then from the three provinces of this region, KBA province was chosen randomly. Among the five IAU universities in KBA province, Gachsaran and Yasuj Universities had between two to three classes of about 30 to 40 Junior Nursing students in each semester. So, three out of the five intact classes in these universities were chosen by the fish-bowl method. Subsequently, they were randomly assigned to the three groups of the study, i.e., PPBL = 30, HPBL = 30 and COTL = 35. These students were of the same socio-economic status, ethnic background and common abilities [44]. These classes were tested for homogeneity through comparing their scores on PN I and Prior Performance Test.

### Instruments of the study

The Pediatric Nursing Performance Test (PNPT) and The Paas Mental Effort Rating Scale (PMER) were utilized as the instruments. In addition, the two-Dimensional Instructional Efficiency Index (IEI) was used to calculate the instructional efficiency. The PNPT comprised 22 multiple-choice items in order to assess the lower-order cognitive domain performance and 22 open-ended questions to determine the higher-order cognitive domain performance. One point was assigned to each correct answer of the multiple-choice items and three points to the open-ended questions. Therefore, the overall performance test for the PNPT ranged between 0 and 88. The higher-order cognitive domain performance referred to students’ ability to analyze, synthesis, and evaluate the PN problems, while the lower-order cognitive domain performance referred to students’ ability to recall, comprehend, and apply [45, 46] the PN problems. Hence, the total scores for low and higher-order cognitive domains were 22 and 66, respectively.

The PMER was used to measure the cognitive load by recording the perceived mental effort which was exerted to solve the problems given during the experiments. This is a 9- point symmetrical Likert scale measurement through which subjects rate their mental effort used in performing a special learning task [47]. The numerical values and labels assigned to the categories ranges from very, very low mental effort (1) to very, very high mental effort (9). Moreover, two kinds of subjective rating of mental effort were also employed. Once, the subjective rating of mental effort was given during the learning in the assessment phase for each main topic. Once more it was given during the test phase. The mental effort per problem or question was obtained by dividing the perceived mental effort by the total number of problems tried at each assessment phase during the learning and the test phases.

Moreover, the two-dimensional (two-D) IEI [48] was applied to compare the effects of different instructional conditions on learning as instructional efficiency. For this purpose, the grand mean was subtracted from each score and the result was divided by the overall standard deviation yielding corresponding z-scores for effort (R) and performance (P). Finally, a performance efficiency score, E, was computed for each participant using the formula E = [(P-R)/2^½^]. High efficiency was indicated by a relatively high test performance in combination with a relatively low mental effort rating. In contrast, low efficiency was indicated by a relatively low test performance in combination with a relatively high mental effort rating.

### Pilot study

In this study, a pilot study was conducted to validate the experimentation procedures and process. Besides, the instruments used in the experiments were validated, and their reliability was ascertained. Besides, some experimental evidence of the appropriateness of the Pediatric Nursing topics in the learning area of organ dysfunction was provided. It also presented a sound basis for structuring the practice and evaluation phases incorporating PBL strategy in the teaching and learning of organ dysfunction in Pediatric Nursing.

In the pilot study, the researcher offered the same topics of Pediatric Nursing to a group of 40 junior nursing students of one intact class who were not included in the actual study. Then the questions on the topics were given to the students to assess their mental efforts. This enabled the researcher to evaluate the validity, reliability, item difficulty, clarity, and feasibility of the test items for the experiment. The Content Validity Ratio (CVR) was (0.99), the reliability of the multiple-choice tests were 0.81 (KR 20 was used), and the reliability of the open-ended questions were 0.88 (Cronbach’s alpha was used). In addition, item difficulty and discrimination indices were shown to be appropriate, ranging from .32 to .70 and .92 to .94, respectively. In addition, using Cronbach’s alpha, the reliability of the mental effort for lower-order questions, higher-order questions and the total questionnaire were calculated to be 0.92, 0.94, and 0.96, respectively.

### Training curriculum

The PN core course is given to junior nursing students. It focuses on caring for children suffering from various physiological and system disorders. Its content consists of nine main topics about disturbance or dysfunction in different systems or organs and their nursing care, from which the four high prevalent conditions such as disturbance of fluid and electrolytes, renal dysfunction, respiratory dysfunction, and gastrointestinal dysfunction were selected for intervention [49, 50].

The course plans, lesson plans, scenarios and Patients Information Sheets (PISs), and tests to provide the general guidelines for teaching the topics on PN for conventional, PPBL, and HPBL strategies, and timetable for all the groups were prepared in advance. Each set of the lesson plans involved five phases of induction, acquisition, practice, closure, and assessment.

In PPBL and HPBL, each group had a leader and a scriber to manage the group activities and take note of their performance, respectively. For the PPBL group, the facilitator started the induction phase by delivering the trigger to the students at the introduction of each main topic for about 5–10 min. He continued the rest of the sessions by asking the learners to recall the materials learned and presenting the information about the related problem to the class. In the acquisition phase, the facilitator managed to give some PISs to the students based on their needs. Then the students detected the concepts by inquiry, self-directed learning and group discussion, worked on trigger to separate known from unknown issues, extracted learning issues during the brainstorming, did research to find the target unknown variables, and recognized all the resources and information they needed to solve the problem. Next, they figured out the best way to achieve the main goal based on the information gathered (hypotheses generation process) and generate the most viable solutions. In the following sessions, the results of each individual’s search had to be communicated to the group members so that the data could be analyzed and incorporated into the process of problem solving. After several cycles of data collection and analysis, possible solutions to the problems were generated and formulated. Finally, the students examined the outcome of their solutions. This general subject presentation took for 75–95 min depending on the sessions and the methods that were used.

In the practice phase, the facilitator reinforced the understanding of the students by discussing the similarities and differences among PISs, eliciting performance and justification of the decision made in about 5 min. In the closure phase, the important concepts about the scenario were highlighted or presented by the students in about 10–15 min. After each main topic, the assessment questions were given to the students to process, work and present the problems individually, and discuss them with the whole class during 10 min.

For the HPBL group, the induction phase started with an example related to the real world problems of concern to the learners so that they could take ownership of their learning. For example, a picture of a child with sunken eyes was first showed to the students for 5–10 min as a trigger to gain their attention. Then some general subjects such as biological development, assessing child with highlighting the differences between children and adults for each specific organ dysfunction with some examples were presented and discussed through mini-lectures in 10 min in conjunction with prior knowledge and scenarios. Moreover, at the end of each HPBL lesson, a short corrective feedback session and summarization of about 15 min were provided by the lecturer. The rest of the specific topics were, however, presented as in the PPBL strategy through triggers under the guidance of the facilitator.

In the control group, the lecturer informed the students of the objectives of the lesson and gave them an example to gain their attention and activate their prior knowledge. In next sessions, the facilitator started the induction phase by asking the students to recall the materials learned and continued to lecture in the acquisition phase in a whole-class instruction by explaining the PN concepts and discussing the steps of processing nursing problems related to the acquired concepts. In the practice phase, the students were asked to process the problem individually and highlight the important aspects. Then, the lecturer handled the discussion of problem solving and made some possible conclusions of the lesson. Finally, at the end of each main topic, each student was required to assess and solve the problems using paper and pencil method. The content and the format of the assessment were the same for all the groups, nonetheless (see Additional file 1).

### Procedures and data collection

This study was conducted during the first semester of the 2010/11 academic year. First, after assigning the participants to three groups, a general briefing session about the strategy was held, and then the training sessions on PPBL and HPBL strategies for two experimental groups were run. Further, it was explained and illustrated to all the students how to use the PMER. After that, the experimental groups received teaching on organ dysfunction in Pediatric Nursing through the PPBL and HPBL strategies for 8 wk while the control group was exposed to only conventional whole-class instruction. All the three groups had similar conditions in terms of the content materials and contact hours. Altogether, 4 main topics were covered during 12 two-hour sessions in the study. After covering each of the four topics, the students were assessed for their learning rate of the contents. They were also required to rate the amount of mental effort they exerted for each problem right after each question.

**Fig. 1:**
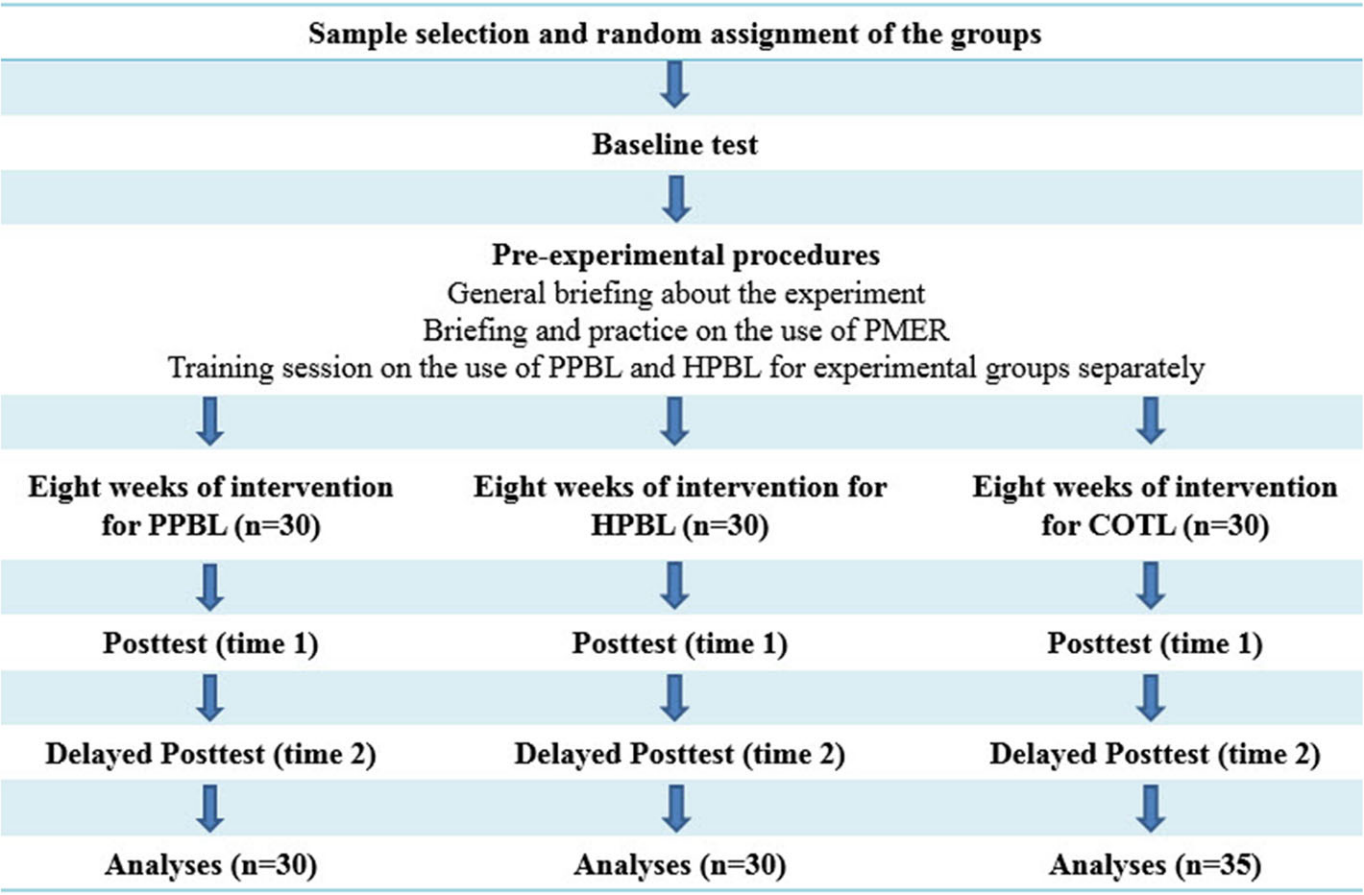
Provides a summary of all the procedures from sample selection to analyses of the findings undertaken in this study

At the end of the experiment, the PNPT and PMER were administered. Next, the Instructional Efficiency was estimated by two-Dimensional IEI. For each question in PNPT, the PMER was printed at the end of the question. After each question, the students were required to indicate the amount of mental effort invested for that particular question by responding to the nine-point symmetrical scale. Finally, a delayed posttest was administered 8 wk after the experiment to assess their retention of the materials [51]. The delayed posttest included the PNPT with PMER. After administering the tests, instructional efficiency was measured using two-Dimensional IEI Formula. Figure 1 provides the flow chart of the study protocol.

Since decay and interference concerning the posttest and delayed posttest were assumed to be equally distributed phenomena in the conventional and PBL groups, they did not serve as covariates in the experiment. Accordingly, if the experimental groups’ learning remains even after the 8 wk elapse, it could be concluded that the effects of strategies performed for experimental groups were more effective in the retention of learning than those of the conventional strategy.

### Data analysis

Using SPSS software version 17, the parametric statistical tests such as ANCOVA, One-way ANOVA, and Mixed between-within subjects ANOVA were used. For these statistical tests with power analysis of Compromise method, with the effect size (ES) = 0.25 which is close to medium, and β/α ratio of 1:1 that is also the default in basic research, and for a total sample size of 95 for 3 groups, the Power was calculated to be more than 0.80. It should be pointed out that alpha level for data analysis was set at 0.05.

### Ethical issues

To carry out this study, same official permissions were taken from the university chancellors. It should be mentioned that the researchers were not allowed to reorganize the student population of the classes. However, some certain measures were taken not to treat the participants unfairly. First, the assignment of the groups to the methods of intervention was purely random. Second, the same amount of materials and time was provided for all the three groups. In addition, written permission to use the instruments in this study was attained from the developer before using them.

## Results

Table 1 offers the demographic background information of the participants. All the students were nursing major and female. Their age ranged from 21 to 25 with a mean of 22.22. A majority (84.21%) of them were from the age group 21–23, whilst the rest (15.79%) were above 23.

**Table 1:** Background information of the participants

Background information	PPBL *n* = 30	HPBL *n* = 30	COTL *n* = 35
Gender	Female	Female	Female
School	Gachsaran	Gachsaran	Yasuj
Age (mean ± SD)	22.30 ± 1.21	22.13 ± 1.33	22.23 ± 1
Age (range)	21–25	21–25	21–24

The result of ANOVA in the three groups of PPBL, HPBL and COTL showed that there were no significant difference between the mean of Pediatric Nursing I scores (F (2, 92) = .34, *p* > .05), and the mean of Prior Performance Test scores (F (2, 92) = .096, *p* > .05).

The exploratory data analysis confirmed that the data conformed to the preliminary assumptions of the parametric tests. The relationship between each dependent variable with its covariate was linear, thus the assumption of a linear relationship was not violated, hence the power of the test. Based on correlation matrix, there was a strong, positive correlation (*r* = .71, *r* = .84, and *r* = .76, *n* = 95, *p* < .0005) between the higher-order performance, the lower-order performance, and the total performance in posttest and their related covariate, respectively. The results indicated that the assumption of homogeneity of regression slopes was not violated for each of the dependent variables and their respective covariates.

On the other hand, the ANCOVA test analysis showed that there were significant differences between the three intervention groups on the overall and the higher-order performances at posttest, with a large effect size [52]. The Tukey HSD test indicated that the means of overall and the higher-order performances for COTL group were significantly lower than those for the PPBL and HPBL groups. But, there was no significant difference between the three intervention groups on the lower-order performance (Table 2).

**Table 2:** Means, SDs, and ANCOVA for Overall, Higher-order, and Lower-order Performances on Posttest

Measures	Groups	n	Mean	SD	F	df	p	η^2^
Overall Performance	PPBL	30	56.10	9.56				
	HPBL	30	56.70	9.44	14.32	2.91	.001	0.24
	COTL	35	49.04	8.91				
Higher-order Performance	PPBL	30	39.60	7.97				
	HPBL	30	40.04	7.20	17.23	2.91	.001	0.28
	COTL	35	32.56	7.36				
Lower-order Performance	PPBL	30	16.50	2.27				
	HPBL	30	16.67	2.99	.078	2.91	.925	0.002
	COTL	35	16.49	2.27				

The ANOVA test analysis showed that there were significant differences between the three intervention groups in the mean of mental effort during the learning phase test, the mean of mental effort during the posttest phase, and the mean of instructional efficiency with moderate to a large effect size. The Tukey HSD test indicated that the means of mental effort during the learning phase test and the mental effort during the posttest for the COTL group were significantly higher than those of the PPBL and HPBL groups. Besides, the mean score of instructional efficiency of the COTL group was significantly lower than those of the PPBL and HPBL groups (Table 3).

**Table 3:** Means, SDs, and ANOVA for Mental Effort of assessment test, and Mental Effort and Instructional Efficiency of the Posttest

Measures	Groups	N	Mean	SD	F	df	P
Mental Effort of assessment test	PPBL	30	3.86	1.35			
	HPBL	30	3.71	1.16	5.67	2.92	.005
	COTL	35	4.75	1.52			
Mental Effort of posttest	PPBL	30	3.80	1.01			
	HPBL	30	3.64	.87	13.69	2.92	.001
	COTL	35	4.89	1.23			
Instructional Efficiency	PPBL	30	.38	1.23			
	HPBL	30	.52	1.12	10.88	2.92	.001
	COTL	35	−.77	1.34			

The ANOVA test analysis showed that there were significant differences in the mean of the overall, the higher-order and the lower-order performances, the mental effort and the mean of instructional efficiency of the three intervention groups in the delayed posttest phase, with the effect sizes of .42, .50, .14, .20, and .31, respectively. The Tukey HSD test indicated that the mean of overall, and higher-order performances, and instructional efficiency for the COTL group were significantly lower than those for the PPBL and HPBL groups. Also, Post-hoc comparisons indicated that the mean score of lower-order performance of the COTL group was significantly lower than that of the HPBL group. However, the PPBL group did not differ significantly from the HPBL and COTL groups on their lower-order performance. Moreover, it was shown that the COTL group invested mental effort significantly more during the delayed posttest than the other groups (Table 4).

**Table 4:** Means, SDs, and ANOVA for Overall, Higher-order, and Lower-order Performances, Mental Effort and Instructional Efficiency, on Delayed Posttest

Measures	Groups	n	Mean	SD	F	df	P
Overall Performance	PPBL	30	30.70	9.32			
	HPBL	30	32.95	8.68	32.82	2.92	.001
	COTL	35	17.89	6.47			
Higher-order Performance	PPBL	30	21.97	7.28			
	HPBL	30	22.85	6.09	45.39	2.92	.001
	COTL	35	10.14	4.76			
Lower-order Performance	PPBL	30	8.73	2.30			
	HPBL	30	10.10	2.82	7.62	2.92	.001
	COTL	35	7.74	2.16			
Mental Effort	PPBL	30	4.20	1.42			
	HPBL	30	4.09	1.56	11.23	2.92	.001
	COTL	35	5.58	1.30			
Instructional Efficiency	PPBL	30	.48	1.22			
	HPBL	30	.68	1.26	20.94	2.92	.001
	COTL	35	−.99	.98			

The statistics presented in Table 5 showed a significant main effect in the mean test of overall and higher-order performances, mental effort and instructional efficiency of the type of instructional strategy, with large effect sizes. The mean scores of the students’ overall and higher-order performance and instructional efficiency were significantly higher in the PPBL and HPBL strategies than in the COTL strategy. The mean scores of the students’ mental effort were significantly lower in the PPBL and HPBL strategies than in the COTL strategy. However, the main effect of the mean score of the lower-order performance based on the type of instructional strategies was insignificant with a small effect size.

**Table 5:** Mixed Between-Within Subjects ANOVA of Overall, Higher-order, and Lower-order Performances, Mental Effort and Instructional Efficiency over Repeated Measures

Measures	Groups	SS	df	MS	F	p	η^2^
Overall Performance	Between-subject effects Groups (G)	5074.633	2	2537.316	17.216	.000	.272
	Error	13,559.313	92	147.384			
	Within-subject effects Times (T)	33,861.306	1	33,861.306	6253.491	.000	.986
	T × G	498.945	2	249.472	46.072	.000	.500
	Error	498.160	92	5.415			
Higher-order Performance	Between-subject effects Groups (G)	4227.334	2	2113.667	23.838	.000	.341
	Error	8157.321	92	88.667			
	Within-subject effects Times (T)	17,197.76	1	17,197.76	3850.91	.000	.977
	T × G	278.286	2	139.143	31.157	.000	.404
	Error	410.862	92	4.466			
Lower-order Performance	Between-subject effects Groups (G)	52.316	2	26.158	2.521	.086	.052
	Error	954.452	92	10.374			
	Within-subject effects Times (T)	2795.680	1	2795.68	1497.89	.000	.942
	T × G	38.259	2	19.129	10.249	.000	.182
	Error	171.710	92	1.866			
Mental Effort	Between-subject effects Groups (G)	75.56	2	37.78	13.08	.000	.22
	Error	265.83	92	2.89			
	Within-subject effects Times (T)	12.28	1	12.28	46.53	.000	.34
	T × G	.78	2	.39	1.48	.232	.03
	Error	24.28	92	.26			
Instructional Efficiency	Between-subject effects Groups (G)	87.52	2	43.76	15.80	.000	.26
	Error	254.83	92	2.77			
	Within-subject effects Times (T)	.007	1	.007	.08	.781	.001
	T × G	1.43	2	.71	8.06	.001	.15
	Error	8.15	92	.09			

The within-subjects effect data also indicated that there were significant differences in the mean scores of overall, higher-order, and lower-order performances, and mental effort across the two time periods with large effect sizes. However, the within-subjects effect data indicated that there was not a significant difference in the instructional efficiency mean scores across the two time periods. In other words, the instructional efficiency did not differ over times.

Further, it was found that there was also a significant interaction effect between the times and instructional strategy group for overall, higher-order, and lower-order performances and instructional efficiency. It was observed that the magnitude of the differences in the means was large, indicating that the overall, the higher-order, and the lower-order performances, and the instructional efficiency depended on both instructional strategy and times of posttest and delayed posttest. However, it was found that there was not a significant interaction effect between the times and instructional strategy group for mental effort. There was a substantial main effect for time with three groups, showing an increase in mental effort test scores across the two time periods.

**Fig. 2:**
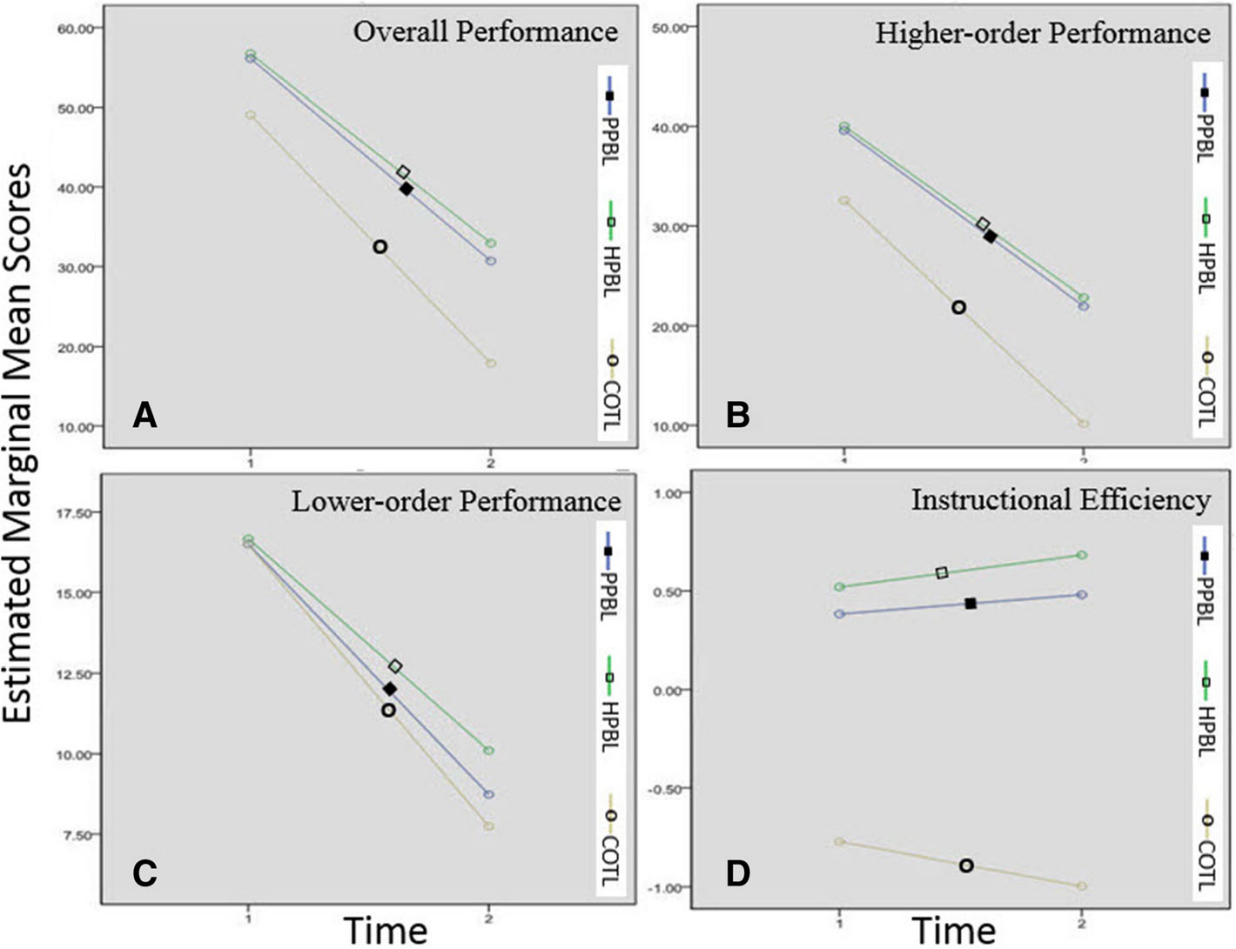
Provides the interaction between the times (1 = posttest and 2 = delayed posttest) and the instructional strategy type for overall, higher-order, and lower-order performances and instructional efficiency based on estimated Marginal Mean Scores over Repeated Measures on Posttest and Delayed Posttest

Figures 2a, b, and c depict the interaction between the times and the instructional strategy type for overall, higher-order and lower-order performances. It was observed that there were general decreases in the overall, higher-order and lower-order performances across the two times for the three groups. The rates of the decreases were greater for the COTL group from posttest to delayed posttest than for the PPBL and HPBL strategy groups. It is interesting to mention that the conventional strategy was less effective than the other strategies in terms of information retention. The interaction might also be explained by the large F value in ANOVA.

Figure 2d depicts the interaction between the times and the instructional strategy type for instructional efficiency. It can be observed that as time increased, the amount of instructional efficiency index of the COTL strategy decreased while, for PBL strategies, there was a general increase in the instructional efficiency indices across the two times for the two groups. Thus, the conventional strategy proved to be less effective than the PPBL and HPBL strategies. The interaction may also be explained by the large *F* value in ANOVA.

Furthermore, pairwise comparison tests were utilized to compare mean differences of the students’ overall, higher-order, and lower-order performances, and instructional efficiency indices. The statistics in Table 6 indicates that there were no significant differences in the obtained mean difference scores of the PPBL and HPBL groups (*p* > .05). However, there were significant differences in the obtained mean differences of overall and higher-order performances and instructional efficiency indices of the COTL strategy with the PBL groups (*p* < .0001). The data analysis indicates that there was not a significant difference in the obtained mean difference of lower-order scores of the PPBL and the other two groups, i.e., the HPBL and COTL groups (*p* > .05). However, there was a significant difference in the obtained mean difference of lower-order scores of the COTL strategy and the HPBL group (*p* = .028).

**Table 6:** Pairwise Comparisons Test of Overall, Higher-order, and Lower-order Performances, and Instructional Efficiency Indices for Groups over Repeated Measures

(I) Group (Instructional Strategies)		(J) Group (Instructional Strategies)	Mean Difference (I-J)	Std. Error	Sig.^a^
Overall Performance	PPBL	HPBL	−1.427	2.216	.521
		COTL	9.936^*^	2.136	.000
	HPBL	PPBL	1.427	2.216	.521
		COTL	11.362^*^	2.136	.000
	COTL	PPBL	−9.936^*^	2.136	.000
		HPBL	−11.362^*^	2.136	.000
Higher-order Performance	PPBL	HPBL	−.6600	1.71917	.922
		COTL	9.4333^*^	1.65664	.000
	HPBL	PPBL	.6600	1.71917	.922
		COTL	10.0933^*^	1.65664	.000
	COTL	PPBL	−9.4333^*^	1.65664	.000
		HPBL	−10.0933^*^	1.65664	.000
Lower-order Performance	PPBL	HPBL	−.767	.588	.196
		COTL	.502	.567	.378
	HPBL	PPBL	.767	.588	.196
		COTL	1.269^*^	.567	.028
	COTL	PPBL	−.502	.567	.378
		HPBL	−1.269^*^	.567	.028
Instructional Efficiency Indices	PPBL	HPBL	−.1695	.30386	.843
		COTL	1.3153^*^	.29281	.000
	HPBL	PPBL	.1695	.30386	.843
		COTL	1.4848^*^	.29281	.000
	COTL	PPBL	−1.3153^*^	.29281	.000
		HPBL	− 1.4848^*^	.29281	.000

*The mean difference is significant at the .05 level

^a^Adjustment for multiple comparisons: Bonferroni

## Discussion

The main purpose of this experiment was to investigate the effects of the PBL strategies on performance, mental effort, and instructional efficiency in learning the organ dysfunction in Pediatric Nursing course. The results showed that the PBL strategy groups had superior overall and higher-order performances compared to the COTL strategy group on posttest and delayed posttest. The implication is that the PBL strategies could be a more effective means than the COTL strategy to maintain students’ performance over time.

Findings also confirmed the earlier studies which were conducted on PBL approach in nursing and in different disciplines [21, 22, 37, 39, 41, 53–56]. For example, Tiwari [39] and Strobel and Berneveld [57] noted that the consensus of their study was that nursing students who were exposed to PBL strategies displayed worthwhile and profound improvement in learning and long-term retention. Chou and Chin [58] also observed that the students in PBL strategy performed better in higher-order thinking performance than the control group. In addition, some researchers found that the structure and process of PBL programs enhanced students’ critical thinking and prepared them to meet the entry-to-practice competencies [53]. A study done by Murphy et al. [54] also indicated a positive effect of PBL strategy on students’ awareness of creative thinking and decision-making abilities. Furthermore, the findings of the present study concurred with the study done by Sangestani and Khatiban [22], who proved that PBL students had a higher performance and learning progress as compared to the COTL group in midwifery. Another study revealed that implementing PBL in educational setting can raise the likelihood of conceptual change by attending lecture-based and self-study groups [21]. However, PBL was not found to be significantly effective in improving critical thinking in some other studies [59]. This is perhaps due to different educational methods, learning environments, and the processes or length of interventions [60], students’ and educators’ attitudes, and cultural backgrounds that can bring different educational effects [61].

In consistent with some other studies [38, 40, 41, 57, 62, 63], the present paper revealed that there was not a significant difference in lower-order performance between the PBL and the COTL instruction group on the posttest. One reason might be that PBL has been designed to promote high-order performance and stimulate students’ higher level skills such as critical thinking [53, 64, 65]. Another reason could be that the learners were only familiar with the conventional instruction of lecturing, and they were, therefore, unable to uncover information without the help of their instructor. One more reason might be due to the split attention effect resulted from several activities. The students were required to refer to different and vast sources and attend to different activities in the PBL groups [66–68]. Hence, lower-order performance was hindered during learning. It could also be due to the anxiety and the low confidence level that the students, especially the weaker ones, tend to experience at the beginning of the PBL strategy [62, 69]. Therefore, it can be argued that a mixed or combined approach of PBL might work better than PPBL.

A remarkable finding related to the retention period was that the PBL students gained slightly less knowledge, but elaborated on and remembered more of the acquired knowledge [41, 70–72]. Likewise, our findings, in agreement with Beers and Bowden [73], showed that although there was not a significant difference in lower-order performance among the groups on the posttest and between the PPBL and COTL on the delayed posttest, the HPBL group had better scores on lower-order performance as compared to COTL group on the delayed posttest. One possible explanation could be attributed to the attention on elaboration in HPBL which tends to promote the recall of declarative knowledge. The implication is that HPBL strategy could be a more effective means than COTL strategy to prevent from a drastic decrease in students’ lower-order performance over time (posttest and delayed posttest).

The results also showed that the performance levels of the PBL strategy groups were achieved with a lower mean mental effort during the learning and test phases. They also suggested that the higher performance was achieved with a reduction in cognitive load (mental effort). These findings were supported by the significantly higher level of two-D instructional condition efficiency index reported by the PBL instructional strategy groups as opposed to the COTL strategy group. Therefore, the study yielded additional empirical evidence in support of HPBL strategy in learning Pediatric Nursing contents.

The results of this study also revealed that the PBL groups achieved better performance on the posttest and delayed posttest. In line with the predictions made by the cognitive load theory, the PBL strategies reduced the learners’ mental effort during learning activities, hence allowing for an increase in overall performance. This contention was further supported by the significantly higher level of two-D instructional condition efficiency index reported by the PBL strategy as opposed to the COTL strategy. There is still more research evidence indicating that PBL increases conceptual change in learners through engaging them cognitively in problem-solving tasks, involving them in group discussions, activating their prior knowledge and providing them with enough opportunity to be able to deeply process the information at hand [21].

The findings of the study further support the contention that cognitive load theory is a useful tool in designing efficient instruction. These findings are congruent with an earlier study conducted by McMullan, Jones and Lea [74] reporting the effect of an interactive e-drug calculations package on nursing students’ drug calculation ability and self-efficacy. As Harasym, Tsai and Munshi [75] and Yuan et al. [5] indicated nursing/medical students in PPBL strategy experienced a heavy workload, had difficulty to search for information and develop tasks and hand-outs, felt too stressed, had difficulty to catch the key points, grasped knowledge incompletely, received vague information and lacked confidence in clarifying the information. In answer to the necessity raised by these researchers, the current study used a combined instructional strategy (HPBL) to minimize working memory overload and decrease students’ split attention for better performance [37, 62, 75].

One reason for the significantly better performance of the PBL groups could be that the PBL strategies had the effect of increasing germane cognitive load. In line with the PBL strategies, the learners were encouraged to analyze, interpret, guess and hypothesize meaning. Moreover, the students were required to link new ideas with previous knowledge, use open-ended questions to promote learners’ participation in class discussion and keep themselves active in the process of learning. In other words, the most important tasks of the tutors were stimulating active and self-directed learning [76]. Also, the researchers, by facilitating the communication among the group members, caused the distribution of cognitive load among the members of the group, fostered learning and increased germane cognitive load through construction of schemata. Therefore, the extraneous or ineffective cognitive load was minimized.

In PBL strategies, the tutors manage to guide the learners through the instructional help in order to reduce the cognitive load by controlling and decreasing the extraneous load and increasing the germane load [25, 48]. Doing so, the learner can solve the problems in need of high mental effort with less effort. This study is in concordance with Martenson et al. [77] who showed that PBL learners’ long-term retention rate was higher than that of the conventional learners. The reason could be that distributing learning over time in PBL strategy typically benefits long-term retention [78]. Generally, the results of the current experiment support the contention that the use of PBL strategies in the learning of Pediatric Nursing course can reduce cognitive load and lead to better overall and higher-level thinking performance.

Another alternative explanation for better performance among the PBL groups could be the design of PBL instructional strategy which is based on constructivism and Vygotsky’s theory. In this study, by interaction between the learners and the facilitator in class as a social context, and through guided instruction that increased interaction between them, the students were able to understand and learn the content materials more quickly and more correctly in the zone of proximal development through more effective instruction [34]. One reason could be that through guidance, scaffolding takes shape and develops sooner to enable students to “bridge the gap between their current abilities and the intended goal” [35]. It is essential to note that the need for a structured and comprehensive feedback and guidance for PBL facilitators has been considered vital in PBL related research [79, 80]. Similarly, this study supports the role of the facilitator as a guide or coach to encourage learners’ progress. Moreover, use of real-world examples, collaborative learning, and class discussions allow facilitators to maintain a sense of control and transition to constructivist teaching [81]. In addition, instructional designers should develop different instructional models and resources that could create a constructivist environment [82].

The present findings highlight the static form of instruction used in the conventional method of teaching Pediatric Nursing. Instructor-based teaching, that requires students to study and then recite what they have already learned, encourages them to merely memorize the textbook contents. It was also observed during the experiment that the students in the conventional strategy group played a more passive role where they were expected to be told what to do and look for the right answers. Dunkin and Biddle [83] also support the notion that students, as a context variable, need to be considered in the teaching and learning process. They also add that passivity among students often induces lower learner involvement. However, they suggest that student is not the only element that should be considered; rather, the process of teaching with the use of effective strategies ought to be taken into account.

To make a long story short, the findings of this study was consistent with Dunkin and Biddle [83] model for the study of classroom teaching, Constructive theory, Vygotsky’s theory [14] and Cognitive Load theory (1988). The most significant conclusion that can be drawn from this study is that using PPBL and an intelligent combination of lecture and problem-based learning that may help to facilitate the learning of Pediatric Nursing can result in more effective instruction. As Borhan [84] suggests, Hybrid PBL and PBL approaches should gradually crawl into the academic curriculum in order to develop learners’ ability to solve problems in a large classroom setting [85].

In a word, the PBL strategies prove to be effective in the students’ overall and higher-order achievement. In addition, although the performance of all the groups decreased and their invested mental effort increased over time, the decrease of the performance and the increase of the invested mental effort for the PBL groups were significantly less than those of the conventional group. Therefore, PBL instructional strategies are deemed to be more efficient with better instructional efficiency indices.

### Implications for medical education

The findings of this study revealed that a simple shift in theoretical emphasis from minimal guidance constructivism to guidance in the zone of proximal development leads to a reconceptualization of PBL which is practically in line with the role of facilitator to guide novice learners to fill in the gap [86]. Furthermore, the findings highlighted that integrating cognitive load theory in designing HPBL programs has an innovative effect. From a teaching viewpoint, it is very essential to discover the rules for fostering the germane load. Thus, HPBL was designed based on CLT to ensure that HPBL leads to more knowledge acquisition, better learning transfer, and lower mental effort, hence the efficiency of student learning. In HPBL, gaining learners’ attention before they learn, as well as giving a brief lecture along with an example by the facilitator in the early stages of learning, could reduce irrelevant processing by highlighting essential materials and linking between previous and new knowledge [87].

The primary practical implication of the present study has to do with the instructional strategies. Both PPBL and HPBL strategies created impressive results in students’ performance, mental effort, and instructional efficiency. The findings of this study revealed that the use of PBL strategies could significantly enhance students’ achievement. Therefore, the PBL strategies are preferred over conventional strategy for instruction in Pediatric Nursing.

In line with the above arguments, a major concern for nursing educators could be how to effectively move from a traditional lecture style to a PBL approach. Since the implementation of PPBL is a time-consuming process and needs additional tutors, the HPBL and PPBL with one floating tutor in a large classroom may be adopted to overcome these concerns.

### Limitations

In this study, four main topics of organ dysfunction with high prevalence in Pediatric Nursing syllabus were offered through PBL strategies, and the intervention was done for only 8 wk of teaching sessions. Although the majority of the students benefited from this teaching approach, the results might be suitably generalized only to courses of similar contents and level. In addition, the researchers were not allowed to change the official programs of the universities to randomize the assignment of the subjects to different groups, and the course was obligatory for all the classes, the students did not have the chance to decide to participate in the study; yet, to be fair enough, as it was already mentioned, the assignment of the classes to each approach was purely randomized.

### Suggestions for future research

The current study suggests that incorporating the use of different PBL approaches can be a useful instructional strategy in the teaching and learning of Pediatric Nursing at university level. Therefore, further research is welcome to assess the effectiveness of this approach in other nursing subjects in other instructional contexts to specify its weak and strong aspects.

Furthermore, new research may intend to examine the long-term effects of problem-based learning on students’ higher-level thinking or on using randomized controlled trials to control threats according to different nursing educational settings as noted in Kong et al. [88]. In addition, other studies with a mixed method design could be conducted to reveal richer student perceptions of the problem-based experience. Moreover, the results delivered a much-needed pattern and starting point for educators presenting active learning approaches for Pediatric Nursing courses to build on student knowledge and skills. A combination of PBL and concept map may offer nursing students a variety of ways of learning. Finally, future studies on PBL strategies could involve not only nursing students but also graduated and registered nurses taking part in retraining programs.

## Conclusions

This study established that using PPBL and HPBL in a large classroom setting with one tutor had several positive outcomes. PBL instructional strategies enhanced students’ overall and higher-order performances, induced less mental effort during learning and test phase, and as a result increased two-dimensional instructional efficiency index in learning Pediatric Nursing. Although, there appeared to be no significant difference in lower-order performance among the groups on the posttest, the HPBL group gained higher scores on lower-order performance as compared to the COTL group on the delayed posttest. The implication is that HPBL strategy could be a more effective means than COTL strategy to enable students to continue to enjoy a better lower-order performance over time.

## Additional file

Additional file 1: Major differences among COTL, HPBL, and PPBL. The information in the additional file provides a brief account of some of the major differences like objectives, teacher’s and students’ roles and responsibilities, etc. among the three instructional approaches of COTL, HPBL, and PPBL that were employed in the study. (DOCX 17 kb)

## Abbreviations

COTL: Conventional Teaching and Learning
HPBL: Hybrid Problem-based learning
IAU: Islamic Azad University
IEI: Instructional efficiency index
KBA province: Kohgiluyeh-BoyerAhmad province
PBL: Problem-based learning
PIS: Patient information sheets
PMER: Paas Mental Effort Rating Scale
PN: Pediatric Nursing
PNPT: Pediatric Nursing Performance Test
PPBL: Pure Problem-based learning

## References

[1] Cheraghi M, Salasli M, Ahmadi F (2007). Iranian nurses’ perceptions of theoretical knowledge transfer into clinical practice: a grounded theory approach. Nurs Health Sci.

[2] Kessenich CR, Guyatt GH, DiCenso A (1997). Teaching nursing students evidence-based nursing. Nurse Educ.

[3] Creedy D, Horsfall J, Hand B (1992). Problem-based learning in nurse education: an australian view. J Adv Nurs.

[4] Klunklin A, Subpaiboongid P, Keitlertnapha P, Viseskul N (2011). Thai nursing students’ adaption to problem-based learning: a qualitative study. Nurse Educ Pract.

[5] Yuan HB, Williams BA, Yin L, Liu M (2011). Nursing students’ views on the effectiveness of problem-based learning. Nurse Educ Today.

[6] Dehkordi AH, Heydarnejad MS (2008). The impact of problem based learning and lecturing on the behavior and attitudes of iranian nursing students, a randomized controlled trial. Dan Med Bull.

[7] Aien F, Noorian C (2006). Problem-based learning: a new experience in education of pediatric nursing course to nursing students. Shahrekord Univ Med Sci J.

[8] Bastable SB (2007). Nurse as educator: principles of teaching and learning for nursing practice.

[9] Bengtsson M, Ohlsson B (2010). The nursing and medical students motivation to attain knowledge. Nurse Educ Today.

[10] Tiwari A, Lai P, So M, Yuen K (2006). A comparison of the effects of problem-based learning and lecturing on the development of students' critical thinking. Med Educ.

[11] Quintero GA (2014). Medical education and the healthcare system - why does the curriculum need to be reformed?. BMC Med.

[12] Patel VL, Groen GJ, Norman GR (1993). Reasoning and instruction in medical curricula. Cogn Instr.

[13] Baker CM, Pesut DJ, McDaniel AM, Fisher ML (2007). Evaluating the impact of problem-based learning on learning styles of master’s students in nursing administration. J Prof Nurs.

[14] Vygotsky LS (1978). Mind in society: the development of higher mental processes.

[15] Smagorinsky P (2013). The development of social and practical concepts in learning to teach: a synthesis and extension of vygotsky’s conception. Learn Cult Soc Interact.

[16] DeVries R (2000). Vygotsky, piaget, and education: a reciprocal assimilation of theories and educational practices. New Ideas Psychol.

[17] Brandon AF, All AC (2010). Constructivism theory analysis and application to curricula. Nurs Educ Perspect.

[18] Young LE, Patterson BL (2007). Teaching nursing: developing a student-centered learning environment.

[19] Gagnon GW, Collay M (2006). Constructivist learning design: key questions for teaching to standards.

[20] Nie Y, Lau S (2010). Diffrential relations of constructivist and didactic instruction to students’ cognition, motivation, and achievement. Learn Instr.

[21] Loyens SMM, Jones SH, Mikkers J, van Gog T (2015). Problem-based learning as a facilitator of conceptual change. Learn Instr.

[22] Sangestani G, Khatiban M (2013). Comparison of problem-based learning and lecture-based learning in midwifery. Nurse Educ Today.

[23] Muis KR, Duffy MC (2013). Epistemic climate and epistemic change: instruction designed to change students' beliefs and learning strategies and improve achievement. J Educ Psychol.

[24] Yilmaz K (2008). Constructivism: its theoretical underpinnings, variations, and implications for classroom instruction. Educ Horiz.

[25] Hmelo-Silver C (2004). Problem-based learning: what and how do students learn?. Educ Psychol Rev.

[26] Cónsul-Giribet M, Medina-Moya JL (2014). Strengths and weaknesses of problem based learning from the professional perspective of registered nurses1. Rev Latino-Am Enfermagem.

[27] Qalehsari MQ, Khaghanizadeh M, Ebadi A (2017). Lifelong learning strategies in nursing: a systematic review. Electron Physician.

[28] Staun M, Bergström B, Wadensten B (2010). Evaluation of a pbl strategy in clinical supervision of nursing students: patient-centred training in student-dedicated treatment rooms. Nurse Educ Today.

[29] Barrows HS (1986). A taxonomy of problem-based learning methods. Med Educ.

[30] Dolmans DH, De Grave W, Wolfhagen IH, Van Der Vleuten CP (2005). Problem-based learning: future challenges for educational practice and research. Med Educ.

[31] Camp G (1996). Problem-based learning: a paradigm shift or a passing fad?. Med Educ Online.

[32] Armstrong E, Boud D, Feletti G (1997). A hybrid model of problem-based learning. The challenge of problem-based learning.

[33] Gwee MCE (2009). Problem-based learning: a strategic learning system design for the education of healthcare professionals in the 21st century. Kaohsiung J Med Sci.

[34] Harland T (2003). Vygotsky's zone of proximal development and problem-based learning: linking a theoretical concept with practice through action research. Teach High Educ.

[35] Rosenshine B, Meister C (1992). The use of scaffolds for teaching higher-level cognitive strategies. Educ Leadersh.

[36] Schnotz W, Kirschner C (2007). A reconsideration of cognitive load theory. Educ Psychol Rev.

[37] Hwang SY, Kim MJ (2006). A comparison of problem-based learning and lecture-based learning in an adult health nursing course. Nurse Educ Today.

[38] Shin I-S, Kim J-H (2013). The effect of problem-based learning in nursing education: a meta-analysis. Adv Health Sci Educ Theory Pract.

[39] Tiwari A, Chan S, Wong E, Wong D (2006). The effect of problem-based learning on students' approaches to learning in the context of clinical nursing education. Nurse Educ Today.

[40] Colliver JA (2000). Effectiveness of problem-based learning curricula: research and theory. Acad Med.

[41] Dochy F, Segers M, Van den Bossche P, Gijbels D (2003). Effects of problem-based learning: a meta-analysis. Learn Instr.

[42] Kirschner PA, Sweller J, Clark RE (2006). Why minimal guidance during instruction does not work: an analysis of the failure of constructivist, discovery, problem-based, experiential, and inquiry-based teaching. Educ Psychol.

[43] Choi E, Lindquist R, Song Y (2014). Effects of problem-based learning vs. traditional lecture on korean nursing students’ critical thinking, problem-solving, and self-directed learning. Nurse Educ Today.

[44] Statistical informations of iau. 2009. https://stat.iau.ir. Accessed 10 Apr 2009.

[45] Gronlund NE (2004). Writing instructional objectives for teaching and assessment.

[46] Bloom BB (1966). Taxonomy of educational objectives: the classification of educational goals.

[47] Paas FG (1992). Training strategies for attaining transfer of problem-solving skill in statistics: a cognitive-load approach. J Educ Psychol.

[48] Kirschner F, Paas F, Kirschner PA (2009). Individual and group-based learning from complex cognitive tasks: effects on retention and transfer efficiency. Comput Hum Behav.

[49] Higher Council of Planning in Medical Sciences (2007). Integrated curriculum for nursing students: Curriculum specifications, nursing bachelorette.

[50] Higher Council of Planning in Medical Sciences (2014). Integrated curriculum for nursing students: Curriculum specifications, nursing bachelorette.

[51] Novak JD (2010). Learning, creating, and using knowledge: concept maps as facilitative tools in schools and corporations.

[52] Cohen J (1988). Statistical power analysis for the behavioral sciences.

[53] Applin H, Williams B, Day R, Buro K (2011). A comparison of competencies between problem-based learning and non-problem-based graduate nurses. Nurse Educ Today.

[54] Murphy S, Hartigan I, Walshe N, Flynn AV (2011). Merging problem-based learning and simulation as an innovative pedagogy in nurse education. Clin Simul Nurs.

[55] Tarmizi RA, Bayat S (2010). Effects of problem-based learning approach in learning of statistics among university students. Procedia Soc Behav Sci.

[56] Zhou J, Zhou S, Huang C, Xu R (2016). Effectiveness of problem-based learning in chinese pharmacy education: a meta-analysis. BMC Med Educ.

[57] Strobel J, Barneveld AV (2009). When is pbl more effective? A meta-synthesis of meta-analyses comparing pbl to conventinal classrooms. Interdiscip J Problem-based Lear.

[58] Chou F-H, Chin C-C (2009). Experience of problem-based learning in nursing education at Kaohsiung medical university. Kaohsiung J Med Sci.

[59] Lee J, Lee Y, Gong S, Bae J (2016). A meta-analysis of the effects of non-traditional teaching methods on the critical thinking abilities of nursing students. BMC Med Educ..

[60] Kong LN, Qin B, Zhou YQ, Mou SY, et al. The effectiveness of problem-based learning on development of nursing students’ critical thinking: a systematic review and meta-analysis. Int J Nurs Stud. 2014;51(3):458–69. 10.1016/j.ijnurstu.2013.06.009 23850065

[61] Chan ZC. A systematic review of critical thinking in nursing education. Nurs Educ Today. 2013;33(3):236–40. 10.1016/j.nedt.2013.01.007 23394977

[62] Rowan CJ, McCourt C, Beake S (2008). Problem based learning in midwifery - the students' perspective. Nurse Educ Today.

[63] Beers GW (2005). The effect of teaching method on objective test scores: problem-based learning versus lecture. J Nurs Educ.

[64] Peen TY, Arshad MY (2014). Teacher and student questions: a case study in malaysian secondary school problem-based learning. Asian Soc Sci.

[65] Chan ZC (2012). Role-playing in the problem-based learning class. Nurse Educ Pract.

[66] Cooper G. Research into cognitive load theory and instructional design at UNSW. Sydney: University of New South Wales; 1998

[67] Sweller J (1994). Cognitive load theory, learning difficulty, and instructional design. Learn Instr.

[68] Smith L, Coleman V (2008). Student nurse transition from traditional to problem-based learning. Learn Health Soc Care.

[69] Rowan CJ, McCourt C, Bick D, Beake S (2007). Problem based learning in midwifery - the teachers perspective. Nurse Educ Today.

[70] Gagne ED (1978). Long-term retention of information following learning Frome prose. Rev Educ Res.

[71] Schmidt HG, Nooman HGS ZH, Ezzat ES (1990). Innovative and conventional curricula compared: What can be said about their effects?. Innovation in medical education: An evaluation of its present status.

[72] Wittrock MC (1989). Generative processes of comprehension. Educ Psychol.

[73] Beers GW, Bowden S (2005). The effect of teaching method on long-term knowledge retention. J Nurs Educ.

[74] McMullan M, Jones R, Lea S (2011). The effect of an interactive e-drug calculations package on nursing students' drug calculation ability and self-efficacy. Int J Med Inform.

[75] Harasym PH, Tsai T-C, Munshi FM (2013). Is problem-based learning an ideal format for developing ethical decision skills?. Kaohsiung J Med Sci.

[76] Boelens R, De Wever B, Rosseel Y, Verstraete AG (2015). What are the most important tasks of tutors during the tutorials in hybrid problem-based learning curricula?. BMC Med Educ..

[77] Martenson D, Eriksson H, Ingelman-Sundberg M (1985). Medical chemistry: evaluation of active and problem-oriented teaching methods. Med Educ.

[78] Dunlosky J, Rawson KA, Marsh EJ, Nathan MJ (2013). Improving students’ learning with effective learning techniques promising directions from cognitive and educational psychology. Psychol Sci Public Interest.

[79] Anderson V, Reid K (2012). Students' perception of a problem-based learning scenario in dental nurse education. Eur J Dent Educ.

[80] Mubuuke AG, Louw AJN, Van Schalkwyk S (2016). Utilizing students’ experiences and opinions of feedback during problem based learning tutorials to develop a facilitator feedback guide: an exploratory qualitative study. BMC Med Educ.

[81] Ertmer PA, Newby TJ (2013). Behaviorism, cognitivism, constructivism: comparing critical features from an instructional design perspective. Perform Improv Q.

[82] Herndon VL. Changing places in teaching and learning: a qualitative study on the facilitation of problem-based learning. Minnesota: Capella University; 2016.

[83] Dunkin MJ, Biddle BJ (1974). The study of teaching.

[84] Borhan MT (2012). Problem based learning (pbl) in malaysian higher education: a review of research on learners' experience and issues of implementations. ASEAN J Eng Educ.

[85] Klegeris A, Hurren H (2011). Impact of problem-based learning in a large classroom setting: student perception and problem-solving skills. Adv Physiol Educ.

[86] Dolmans DHJM, Grave WD, Wolfhagen IHAP, Vleuten CPM (2005). Problem-based learning: future challenges for educational practice and research. Med Educ.

[87] Jalani NH, Sern LC (2015). The example-problem-based learning model: applying cognitive load theory. Procedia Soc Behav Sci.

[88] Kong L-N, Qin B, Zhou Y-q, Mou S-y (2013). The effectiveness of problem-based learning on development of nursing students’ critical thinking: a systematic review and meta-analysis. Int J Nurs Stud.

