# Supplementary material for: Pure PBL, Hybrid PBL and Lecturing: which one is more effective in developing cognitive skills of undergraduate students in pediatric nursing course?
Source: BMC Med Educ. 2018 Aug 10;18:195. doi: 10.1186/s12909-018-1305-0 (PMC6086017; doi:10.1186/s12909-018-1305-0)
Supplement: Supplementary file 1 — Major differences among COTL, HPBL, and PPBL. The information in the additional file provides a brief account of some of the major differences like objectives, teacher’s and students’ roles and responsibilities, etc. among the three instructional approaches of COTL, HPBL, and PPBL that were employed in the study. (DOCX 17 kb) [file 12909_2018_1305_MOESM1_ESM.docx]

**Additional File 1: Major differences among COTL, HPBL, and PPBL**

| PPBL | HPBL | COTL | Strategies  Elements |
| --- | --- | --- | --- |
| Problems (defining, hypotheses generation process, and problem solution process) | Subjects, Facts, concepts, and problems (defining, hypotheses generation process, and problem solution process) | Subjects, Facts, concepts | **Main learning objectives** |
| Beginning with a real life problem to get students’ attention (trigger) | Beginning with a real life problem to get students’ attention (trigger) | Starting with cases of real life situation and giving the lesson objectives | **Induction phase** |
| Facilitator | Lecturer (mini) and facilitator | lecturer | **Teacher role** |
| leader, scribe and group member (active and interactive role) | leader, scribe and group member (active and interactive role) | Passive | **Student role** |
| Retrieving previous knowledge and linking with the new problem, self-directed learning and small group discussions | Activating previous knowledge, Learning based on the problem, self-directed learning, and small group discussions | Recite, repeat, practice, reward and punish | **Learning strategies** |
| Lecturer but mainly students | Lecturer and students | Student but mainly lecturer | **Learning responsibilities** |
| Reinforcing student understanding through Q&A on key points | Reinforcing student understanding through Q&A on key points | Individual student answer | **Oral Assessment** |
